# *In Vitro* Activity of Copper(II) Complexes, Loaded or Unloaded into a Nanostructured Lipid System, against *Mycobacterium tuberculosis*

**DOI:** 10.3390/ijms17050745

**Published:** 2016-05-17

**Authors:** Patricia B. da Silva, Paula C. de Souza, Giovana Maria Fioramonti Calixto, Erica de O. Lopes, Regina C. G. Frem, Adelino V. G. Netto, Antonio E. Mauro, Fernando R. Pavan, Marlus Chorilli

**Affiliations:** 1 Faculdade de Ciencias Farmaceuticas, UNESP—Univ Estadual Paulista, Campus Araraquara, Araraquara, São Paulo 14800-903, Brazil; paula_farma85@yahoo.com.br (P.C.S.); giovana.calixto@gmail.com (G.M.F.C.); ericalopes276@gmail.com (E.O.L.); 2 Instituto de Química, UNESP—Univ Estadual Paulista, Campus Araraquara, Araraquara, São Paulo 14800-060, Brazil; rcgfrem@gmail.com (R.C.G.F.); adelino@iq.unesp.br (A.V.G.N.); mauro@iq.unesp.br (A.E.M.)

**Keywords:** copper(II) complexes, tuberculosis, *Mycobacterium tuberculosis*, cytotoxicity, nanostructured lipid system, *Artemia salina* L.

## Abstract

Tuberculosis (TB) is an infectious disease caused mainly by the bacillus *Mycobacterium tuberculosis* (*Mtb*), presenting 9.5 million new cases and 1.5 million deaths in 2014. The aim of this study was to evaluate a nanostructured lipid system (NLS) composed of 10% phase oil (cholesterol), 10% surfactant (soy phosphatidylcholine, sodium oleate), and Eumulgin^®^ HRE 40 ([castor oil polyoxyl-40-hydrogenated] in a proportion of 3:6:8), and an 80% aqueous phase (phosphate buffer pH = 7.4) as a tactic to enhance the *in vitro* anti-*Mtb* activity of the copper(II) complexes [CuCl_2_(INH)_2_]·H_2_O (**1**), [Cu(NCS)_2_(INH)_2_]·5H_2_O (**2**) and [Cu(NCO)_2_(INH)_2_]·4H_2_O (**3**). The Cu(II) complex-loaded NLS displayed sizes ranging from 169.5 ± 0.7095 to 211.1 ± 0.8963 nm, polydispersity index (PDI) varying from 0.135 ± 0.0130 to 0.236 ± 0.00100, and zeta potential ranging from −0.00690 ± 0.0896 to −8.43 ± 1.63 mV. Rheological analysis showed that the formulations behave as non-Newtonian fluids of the pseudoplastic and viscoelastic type. Antimycobacterial activities of the free complexes and NLS-loaded complexes against *Mtb* H_37_Rv ATCC 27294 were evaluated by the REMA methodology, and the selectivity index (SI) was calculated using the cytotoxicity index (IC_50_) against Vero (ATCC^®^ CCL-81), J774A.1 (ATCC^®^ TIB-67), and MRC-5 (ATCC^®^ CCL-171) cell lines. The data suggest that the incorporation of the complexes into NLS improved the inhibitory action against *Mtb* by 52-, 27-, and 4.7-fold and the SI values by 173-, 43-, and 7-fold for the compounds **1**, **2** and **3**, respectively. The incorporation of the complexes **1**, **2** and **3** into the NLS also resulted in a significant decrease of toxicity towards an alternative model (*Artemia salina* L.). These findings suggest that the NLS may be considered as a platform for incorporation of metallic complexes aimed at the treatment of TB.

## 1. Introduction

Tuberculosis (TB) is an infectious and curable disease whose primary etiological agent is *Mycobacterium tuberculosis* (*Mtb*). According to the World Health Organization (WHO), 56 million cases were successfully treated between the years 1995 and 2011, and a 45% reduction in death rate was observed between 1990 and 2012 [1]. However, the WHO report of 2014 recorded 9.5 million new TB cases and 1.5 million deaths. The disease appears to be associated with two factors: (a) the co-infection of *Mtb* with human immunodeficiency virus (HIV) that was responsible for the death of 400,000 people in 2014 and (b) the increasing emergence of strains resistant to drugs commercially available for therapy in the form of multi-drug-resistant TB (MDR-TB) and extensively-drug-resistant TB (XDR TB) that hinder treatment, indicating the need for research on new drugs [2].

The inorganic chemistry of molecules provides great potential in aiding the search for newer drugs to overcome problems related to anti-tubercular therapy. In this context, the introduction of metal centers in the design of novel pharmaceuticals displaying new mechanisms of action represents a stimulating and active field of research [3]. Numerous efforts to develop new metal-based complexes for diagnostic and/or therapeutic purposes have been prompted by the effectiveness of *cis*-diamminedichloroplatinum(II) (*cisplatin*) in the clinical treatment of many tumors [4]. Among metals, much attention has been paid to copper, which is an important trace element for plants and animals [5]. As an essential element, copper complexes may be less toxic than non-essential metals. This has prompted many authors to use copper(II) ions in the design of new bioactive compounds. A literature survey showed numerous Cu(II) complexes with significant antimycobacterial activity against *Mtb* H_37_Rv [6]. For instance, Kanwar *et al.* [7] have described that the copper(II) derivative of isonicotinoyldithiocarbazic acid displays a minimum inhibitory concentration (MIC) value of 2 μg·mL^−1^ against the H_37_Rv strain. Additionally, copper(II) compounds bearing fluorinated isonicotinoylhydrazones and carboxamidrazones have demonstrated an enhanced ability to kill intracellular *mycobacterium* [6,8]. Compounds of the type [CuL_2_(H_2_O)_2_] (L = isonicotinoylhydrazones) have also exhibited promising antitubercular activities against *Mtb* (MIC < 0.2 μg·mL^−1^) [9]. Cu(II) and Zn(II) complexes containing hydrazone derivatives of quinoline as ligand have been evaluated against Mtb and exhibited antimycobacterial activities at concentrations of 6.25 to 25 μg·mL^−1^ [10]. Copper(II) compounds of the type [CuL(X)] (L = Schiff bases obtained by the condensation of 3-hydroxyflavone with 4-aminoantipyrine, *o*-aminophenol, *o*-aminobenzoic acid, *o*-aminothiazole or thiosemicarbazide; X = OH_2_, OAc) have had their growth inhibitory activity investigated against *Mtb* H_37_Rv [11]. The MIC values obtained for the complexes ranged from 1.7 × 10^−3^ to 9.9 × 10^−3^ μg·mL^−1^ and from 10.5 × 10^−3^ to 20.7 × 10^−3^ μg·mL^−1^ for the free ligands. These results show that the coordination of the Cu(II) ion improved the antimycobacterial activity of the ligands.

Our group has been studying copper(II) complexes ([CuCl_2_(INH)_2_]·H_2_O (**1**), [Cu(NCS)_2_(INH)_2_]·5H_2_O (**2**), and [Cu(NCO)_2_(INH)_2_]·4H_2_O (**3**)) containing isoniazid (INH), a drug that is used extensively to treat tuberculosis as ligand; however, the low solubility of metallic complexes in water has hindered the continuity of newer drug research. The use of nanotechnology via incorporation of complexes into the nanostructured lipid system (NLS) has been a useful strategy for improving the solubility of the compounds and consequently increasing the antimicrobial activity against *Staphylococcus aureus* and *Escherichia coli* [12].

Many studies have shown the potential of pharmaceutical nanotechnology in the treatment, prevention, and diagnosis of several pathologies, including TB [13]. The use of nanotechnology for the development of new drugs against tuberculosis aims for (a) protection of the compound in the therapeutic system against possible instability in the organism, promoting the maintenance of plasma levels at constant concentration; (b) increased therapeutic efficacy; (c) the progressive and controlled release of the drug by conditioning the environmental stimuli (sensitive to the pH variation or temperature); (d) the expressive reduction of toxicity by the reduction of peak plasma levels of maximum concentration; (e) reducing the instability and decomposition of sensitive compounds; (f) the possibility of the orientation to specific targets (site specificity); (g) the incorporation of hydrophilic or lipophilic substances in the devices; and (h) the reduction of therapeutic dose and the number of administrations and an increase in patient acceptance of therapy [14].

NLS’s are known to be thermodynamically stable, isotropic, and transparent systems composed of a mixture of oil and water, stabilized by a surfactant film located at the oil/water interface. DDS colloids, such as NLS’s, have many advantages such as increasing the rates of solubility and drug dissolution, protecting unstable molecules from degradation, and promoting a controlled and sustained release [15,16,17,18].

The purpose of this study was to assess an NLS composed of a 10% oil phase (*i.e.*, cholesterol), 10% surfactant (*i.e.*, soy phosphatidylcholine, sodium oleate, and Eumulgin^®^ HRE 40 [castor oil polyoxyl-40-hydrogenated] in a proportion of 3:6:8), and an 80% aqueous phase (*i.e.*, phosphate buffer, pH 7.4) as a tactic to enhance the *in vitro* anti-*Mtb* activity of three copper(II) compounds containing isoniazid (INH) as a ligand: [CuCl_2_(INH)_2_]·H_2_O (**1**), [Cu(NCS)_2_(INH)_2_]·5H_2_O (**2**), and [Cu(NCO)_2_(INH)_2_]·4H_2_O (**3**). The toxicity of these complexes was evaluated on Vero epithelial cells (a normal eukaryotic cell), J774A.1 macrophage, MRC-5 cell lines, and an alternative *in vivo* model of *Artemia salina* L (Artemiidade).

## 2. Results and Discussion

### 2.1. Nanostructured Lipid System

#### 2.1.1. Mean Diameter and Polydispersity Index (PDI)

The diameter of the internal phase of the nanostructured lipid systems was determined via light scattering, and the average values and standard deviation of the droplet size and the polydispersity index are shown in Table 1 for the NLS and the copper(II) complexes incorporated into the NLS.

The average diameter of the droplets of the nanosystem before and after incorporation of the complexes varied between 169.5 ± 0.7095 and 211.1 ± 0.8963 nm; these values are consistent with the characteristic values of NLS’s according to Cunha Junior *et al.* [19]. The NLS’s exhibited PDI values of (0.153 ± 0.0130)–(0.236 ± 0.00100), indicating a high level of homogeneity among the droplets within the NLS.

#### 2.1.2. Zeta (ζ) Potential Analysis

Table 2 provides the zeta potential values for the NLS and copper(II) complexes loaded into the NLS.

The samples showed zeta potential values ranging from −0.00690 ± 0.0896 to −8.43 ± 1.63 mV. This negative charge results from the components of the formulations as described by Oliveira *et al.* [20,21]. The presence of polar substituents in soy phosphatidylcholine and sodium oleate (ester) as well as in cholesterol (hydroxyl), from which the sodium ion can dissociate, contributes to the appearance of negatively charged groups [22].

#### 2.1.3. Rheological Study

**Figure 1:**
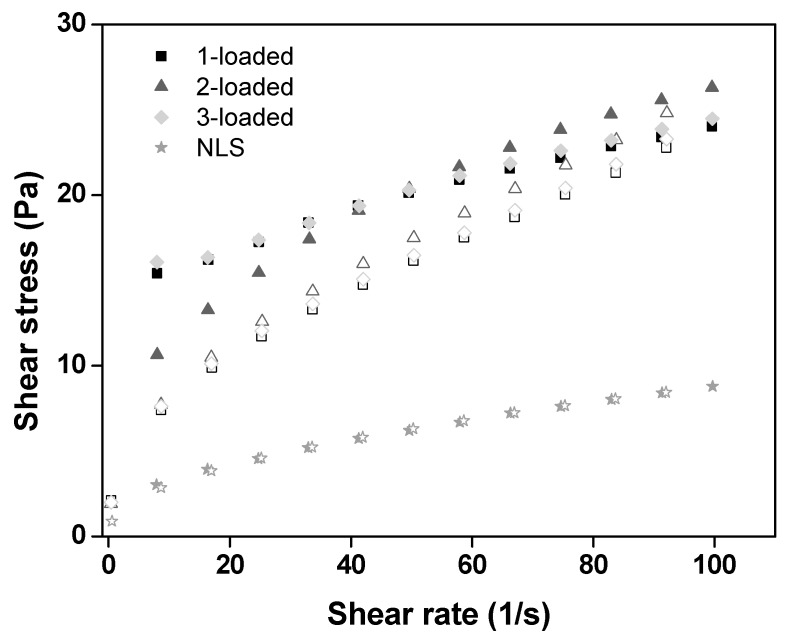
Flow rheograms of NLS, 1-, 2-and 3-loaded into the NLS at 37 ± 0.5 °C. Closed symbol is the going curve and open symbol is the back curve. The standard deviations have been omitted for clarity; however, the coefficients of variation of the triplicate analyses were less than 10% for all formulations.

The rheograms of the formulations are shown in Figure 1, where the relationship between the shear stress (Pa) and shear rate (1/s) demonstrates that all the formulations behaved as non-Newtonian pseudoplastic fluids. From the data obtained through Equation (1) (τ = *k* · γ^η^) and as shown in Table 3, it was certified that these formulations behaved as pseudoplastic fluids.

Such pseudoplastic behavior may result due to the breakage of organized structures on the application of stress, which will thin the flow, thus facilitating the application of the system, which is desirable for pharmaceutical formulations.

Taking into account the K index values (Table 3), it was observed that incorporation of the copper(II) complexes into the NLS increased the consistency index of the formulations, particularly those of 1- and 3-loaded formulations, which could be a result of the interaction of these compounds with the components of the NLS.

The thixotropic of all formulations was also studied. As observed in the rheograms of Figure 1, the formulations containing the complexes (1-, 2-, and 3-loaded) are thixotropic because the outward curve did not overlap with the back curve; whereas, with NLS alone, an overlap of the two curves was observed. This data demonstrates the influence of the complexes on NLS because the presence of different drugs in NLS favored the formation of strong structuration, requiring additional time for the formulations to return to their initial structure [23,24].

**Figure 2:**
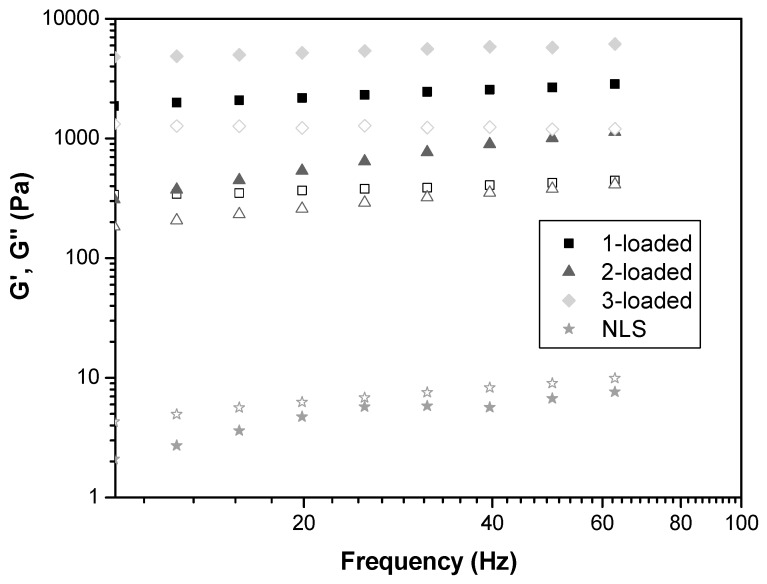
Frequency sweep profiles of NLS, 1-, 2-, and 3-loaded into the NLS at 37 ± 0.5 °C. The storage modulus G′ is the closed symbols. The loss modulus G′′ is the opened symbols. The SDs have been omitted for clarity, but the coefficients of variation of the triplicate analyses were less than 10%.

The oscillatory analysis of the formulations is illustrated in Figure 2. The storage modulus of the 1-, 2-, and 3-loaded formulations was greater than the loss modulus (G’ > G′′), indicating that these formulations have an elastic character. Further, the NLS formulation showed a higher G′′ than G′, implying a viscous character [24].

These findings corroborate data obtained by the continuous rheological analysis and strengthen the observation that the presence of drugs influences the structure of the NLS because they form more organized structures.

Furthermore, 2- and 3-loaded formulations have higher values of G′, indicating that the drug present in such formulations increases the elasticity of the formulation, suggesting that such formulations are structured by stronger secondary chemical bonds [25].

### 2.2. In Vitro Biological Activity

#### 2.2.1. Antimycobacterial and Cytotoxicity Activities

The *in vitro* results (*i.e.*, MIC, IC_50_, and SI values) achieved for the free copper(II) compounds and those loaded into the NLS are displayed in Table 4.

Initially, the MIC values for each compound against *Mtb* H_37_Rv ATCC 27294 were determined using the Resazurin Microtiter Assay (REMA) [26]. The free compounds **2** and **3** were considered active because they exhibited anti-MTB activity at ≤10 μg·mL^−1^ [27].

The three copper(II) compounds containing INH as a ligand in the coordination sphere evaluated in this study were as active against *Mtb* as those complexes synthesized by Bottari *et al.* containing a ligand derivative of isoniazid [9].

However, upon the loading of the copper(II) complexes into the NLS, a significant enhancement of MICs was observed for all compounds—from 20.46 to 0.397 μg·mL^−1^ for compound **1**, 5.913 to 0.219 μg·mL^−1^ for compound **2**, and 1.459 to 0.313 μg·mL^−1^ for compound **3**. Therefore, a 52-, 27-, and 4.7-fold increase in activity was reported for these compounds, respectively.

Our studies show that formulations with a high degree of structuring increase their stability and retention in the biological membrane, permitting a longer duration of the drug at the target site. This improvement in the biological activity can thus be explained by rheological parameters because all complex-loaded NLS’s present a high consistency index and elasticity, thereby forming a more structured formulation with the complex incorporation [20,28,29].

Furthermore, such enhancement in the biological activity can also be described by the appearance of thixotropy for 1-, 2-, and 3-loaded NLS’s, as these formulations would require a longer time to return to their initial organization once they are subjected to an applied force and undergo a shear, allowing them to release the drug for longer durations [24,30].

With respect to the cytotoxicity index against Vero cells, the compound **1** unloaded into the NLS showed a lower IC_50_ compared with the other copper(II) complexes and was slightly more toxic. However, it must be noted that all compounds displayed higher IC_50_ values after incorporation into the NLS; in other words, the complexes loaded into the NLS were less toxic. The compound **2** unloaded into the NLS demonstrated more toxicity towards the macrophage cell line (J774A1) compared to the other copper(II) complexes. Analysis of the cytotoxicity results of the copper(II) complexes against the MRC-5 cell line (lung fibroblasts cells) revealed a similar trend in behavior—an increase in toxicity when loaded into the NLS.

This increase in toxicity of the compounds after incorporation into the NLS may be related to greater sensitivity of these cell lines due to a high concentration of lipids that compose the nanosystem. The same behavior was observed for rifampicin (RMP) loaded into the solid lipid nanoparticles (SLNs) containing soy lecithin in their composition. For alveolar macrophages, the cytotoxicity induced by the RMP solution was substantially lower than that induced by the RMP-SLNs [31].

However, in order to gain deeper insights into whether a compound is a promising candidate for the treatment of tuberculosis, it is necessary to evaluate the results of MIC and IC_50_ together via the selectivity index (SI), which is the ratio between the IC_50_ and MIC [32]. Compounds that exhibit an SI ≥10 can be applied with a concentration of 10 or more times the MIC value without showing cytotoxicity. In this study, it was observed that all compounds showed SI values >10 after the incorporation of the complexes into the nanostructured system.

These results showed that the NLS was able to improve the antimycobacterial activity and the selectivity index. The increase in the antimycobacterial activity of the NLS seems to occur at the level of the mycobacterial plasma membrane. The interaction between the NLS and the membrane results in the de-structuration of a phospholipid bilayer, thereby affecting its fluidity, in turn leading to cell death [14,33].

#### 2.2.2. *Artemia salina* L. Toxicity

Bioassays such as *Artemia salina* L. (Artemide), the brine shrimp, can be used to determine the toxicity of compounds over the median lethal concentration (LC_50_) [34]. The data from the LC_50_ of the free copper(II) complexes and those loaded into the nanosystem with *Artemia salina* L. (Artemiidae) are presented in Table 5.

Table 5 shows that compound 3 in dimethylsulfoxide was less toxic against *Artemia salina* L., which is in agreement with the results obtained for the Vero epithelial cells, J774A.1 macrophages, and MRC-5 cell lines (Table 4). After the loading of the copper(II) complexes into the NLS, LC_50_ values changed from 17.10 to 297.7 μg·mL^−1^ (**1**), 7.800 to 378.8 μg·mL^−1^ (**2**), and 244.1 to 479.2 μg·mL^−1^ (**3**). Therefore, the toxicity was reduced by 17-, 49-, and 2-fold for compounds **1**, **2**, and **3**, respectively. The unloaded nanostructured system did not show toxicity against brine shrimp.

Copper(II) complexes solubilized in dimethylsulfoxide investigated in this study were less toxic against the microcrustacean compared to the compound [Cu(2Am4Ph)Cl] (LC_50_ = 2.67 μg·mL^−1^) (2Am4Ph = *N*(4)-phenyl-2-pyridineformamide thiosemicarbazone) reported by Ferraz *et al.* [35].

To the best of our knowledge, there is no report available in the literature regarding the toxicity of coordination compounds loaded into the NLS against the *Artemia salina* L., which makes it difficult to verify the results obtained in this study. However, it was possible to establish a correlation between the Vero cell and the microcrustacean because, for both assays, the compounds not loaded into the nanosystem showed higher toxicity than those loaded into the NLS.

## 3. Material and Methods

### 3.1. Materials

Sodium oleate and cholesterol were purchased from Sigma-Aldrich^®^ (St. Louis, MO, USA). Soy phosphatidylcholine was purchased from Lucas Meyer Gmbh & Co (Hamburg, Germany). Phosphate buffer solution was prepared from sodium monohydrogen phosphate and dihydrogen phosphate that were purchased from Merck (Darmstadt, Germany). Polyoxyl-40 hydrogenated castor oil was purchased from Pharma Special (Itapevi, Sao Paulo, Brazil). Dulbecco’s modified Eagle’s medium (DMEM), fetal bovine serum, gentamicin sulfate, amphotericin B, and trypsin/EDTA were purchased from Vitrocell Embriolife (Campinas, Sao Paulo, Brazil).

### 3.2. Synthesis of the Copper(II) Complexes

The synthesis of the complexes [CuCl_2_(INH)_2_]·H_2_O (**1**), [Cu(NCS)_2_(INH)_2_]·5H_2_O (**2**), [Cu(NCO)_2_(INH)_2_]·4H_2_O (**3**) was performed according to the methodology described by Silva *et al.* [12].

### 3.3. Preparation of Formulations

The NLS’s were prepared as described by Formariz *et al.* and Freitas *et al.* [14,36,37] with the following composition: 10% oil phase (*i.e.*, cholesterol), 10% surfactant (*i.e.*, soy phosphatidylcholine, sodium oleate, and Eumulgin^®^ HRE 40 [castor oil polyoxyl-40-hydrogenated] in a proportion of 3:6:8), and 80% aqueous phase (*i.e.*, phosphate buffer, pH 7.4). The mixture was sonicated using a rod sonicator (Q700 of QSonica^®^, Newtown, CT, USA) at 700 watts in discontinuous mode for 15 min with 30 s incubations in an ice bath every two minutes during the sonication process. After sonication, the NLS’s were centrifuged at 11,180 × *g* for 15 min to eliminate the waste released by the titanium rod sonicator.

The copper(II) complexes were loaded into the NLS by dissolving the compounds in the studied formulation at a concentration of 5000 μg·mL^−1^.

### 3.4. Physicochemical and Structural Characterization of the System

#### 3.4.1. Mean Diameter and Polydispersity Index (PDI)

The NLS droplet size and distribution were determined by an optical particle analyzer (Zetasizer Nano-ZS ZEN3600, Malvern Instruments, San Diego, CA, USA) using dynamic light scattering. All samples (NLS’s with and without inorganic compounds) were diluted in Milli-Q water (100 μL of sample in 900 μL of deionized water) and placed in the instrument. The analyses were performed in triplicate.

#### 3.4.2. Zeta Potential Analysis

The zeta potential (ζmV) was also determined using a dynamic light scattering with a Zeta-Plus (Malvern Instruments, San Diego, CA, USA), where the droplets of the formulation were subjected to a fixed voltage, and the ζmV was calculated using the values provided by the apparatus. Ten measurements of the electrophoretic mobility were made for each sample (*n* = 3).

#### 3.4.3. Rheological Study

Both rheological studies described below were carried out at 37 ± 0.1 °C in triplicate, using a controlled-stress AR2000 rheometer (TA Instruments, New Castle, DE, USA) with steel plate geometry (40 mm diameter) and a sample gap of 200 μm.

Carefully, the formulations were placed to the lower plate in order to decrease the sample shearing. Then, they were allowed to equilibrate for 3 min before the analysis.

##### Determination of Flow Properties

The flow properties were determined using a controlled shear rate procedure ranging from 0.01 to 100 s^−1^ for 120 s and back from 100 to 0.01 s^−1^ for 120 s, as well. The interval between the curves was 10 s. The consistency and flow indices were determined from the power law described in Equation (1) for a quantitative analysis of flow behavior:

τ = *k* · γ^η^ (1)

where “τ” is the shear stress, “γ” is the shear rate, “*k*” is the consistency index, and “η” is the flow index [23,24].

##### Oscillatory Analyses

Firstly, a stress sweep to determine the viscoelastic region of the formulations was performed at a constant frequency of 1 Hz over the stress range of 0–50 Pa. Afterward, the constant shear stress of 1 Pa was chosen to carry out the frequency sweep over a range of 0–10 Hz, which is the linear viscoelastic region for all formulations, in order to record the storage (G′) and loss (G′′) moduli.

### 3.5. In Vitro Biological Activity

#### 3.5.1. *In Vitro* Anti-*Mycobacterium tuberculosis*

The antitubercular activity of the compounds was determined by the REMA methodology as described by Palomino and colleagues [26]. Stock solutions of the tested compounds were prepared in DMSO (Sigma-Aldrich^®^, St. Louis, MO, USA) or in the NLS and diluted in Middlebrook 7H9 broth (Difco/Becton-Dickinson, Franklin Lakes, NJ, USA) supplemented with 10% OADC enrichment (dextrose, albumin, and catalase—BBL/Becton-Dickinson, Franklin Lakes, NJ, USA) to obtain final drug concentration ranges of 0.09–25 μg·mL^−1^. Rifampicin (Sigma-Aldrich^®^) was used as a standard drug. A suspension of the MTB H_37_Rv ATCC 27294 was cultured in Middlebrook 7H9 broth supplemented with 10% OADC and 0.05% Tween 80. The concentration was adjusted to 2 × 10^5^ UFC·mL^−1^, and 100 μL of the inoculum was added to each well of a 96-well microtiter plate together with 100 μL of the compounds. Samples were set up in triplicate. The plate was incubated for 7 days at 37 °C. After 24 h, 30 μL of 0.01% resazurin (solubilized in distilled water) was added. Resazurin is an indicator the cell viability. Resazurin is reduced to resorufin in response to cell respiration, where pink color and fluorescence indicates cell viability. The fluorescence of the wells was read in a Cytation 3 (Biotek^®^, Winooski, VT, USA), in which excitations and emissions filters at wavelengths of 530 and 590 nm were used, respectively. Each test was set up in triplicate.

#### 3.5.2. *In Vitro* Cytotoxic Activity

*In vitro* cytotoxicity assays (IC_50_) were performed on MRC-5 (ATCC^®^ CCL-171), VERO (ATCC^®^ CCL-81), and J774A.1 (ATCC^®^ TIB-67), as described by Pavan *et al.* [38]. MRC-5 is widely used for phenotypic *screening* of drugs and is regarded as a normal cell derived from human lungs, VERO is an epithelial cell from green monkeys, and J774A.1 is a macrophage from carcinoma murine cell. The cells were routinely maintained in complete medium (DMEM (Vitrocell^®^, Campinas, SP, Brazil) supplemented with 10% heat in activated fetal bovine serum (FBS (Vitrocell^®^) plus amphotericin B (2 mg·L^−1^) (Vitrocell^®^) and gentamicin (50 mg·L^−1^) (Vitrocell^®^) at 37 °C, in a humidified 5% CO_2_ atmosphere. After reaching confluence, the cells were detached, counted, and adjusted to 1 × 10^5^ cells·mL^−1^ (for J774A.1 and MRC-5) and 3.4 × 10^5^ cells·mL^−1^ [39]. The cells were seeded in 200 μL of complete medium in 96-well plates (KASVI^®^, Curitiba, PR, Brazil). The plates were incubated under the same conditions for 24 h to allow cell adhesion. Stock solutions of the compounds were diluted to 2-fold serial dilution from 250 to 0.975 μg·mL^−1^. Then, the cells were exposed to compounds at these concentrations for 24 h. Resazurin solution was then added to the cell cultures and incubated for 6 h. The fluorescence measurements (530-nm excitation filter and 590-nm emission filter) were performed in a Cytation 3 (Biotek^®^, Winooski, VT, USA) microfluorimeter. The IC_50_ value was defined as the highest drug concentration at which 50% of the cells are viable relative to the control. Each test was set up in triplicate.

#### 3.5.3. Selectivity Index

The selectivity index (SI) can be calculated by dividing the IC_50_ value by the MIC value. An SI greater than or equal to 10 indicates that the test compound can be applied at a concentration that is ten-fold higher than the MIC value without exhibiting cytotoxicity [32].

#### 3.5.4. *Artemia salina* L. Toxicity

To determine toxicity of the complexes in brine shrimp, 25 mg eggs (Artêmia salina do RN, Natal, RN, Brazil) were incubated in a beaker (2000 mL) with artificial salt water at temperatures ranging from 20 to 30 °C. Artificial salt water consisted of 23 g NaCl, 11 g MgCl_2_·6H_2_O, 4 g Na_2_SO_4_, 1.3 g CaCl_2_·H_2_O, and 0.7 g KCl in 1000 mL distilled water. The pH was adjusted to 9.0 using 5 N NaOH to avoid risk of death to the *Artemia* larvae (nauplii) by decrease of pH during incubation.

After 24 h, 0.6 g of *Saccharomyces cerevisiae* was added to the beaker for every liter of salt water to feed the nauplii; 48 h after the eggs were incubated, the nauplii were extracted for the experiment.

A suspension of nauplii containing 10–15 organisms (120 μL) was added to each well of the 96-well microplates, including the control group. In separate 96-well microplates, serial dilutions of complexes loaded or unloaded into the NLS and DMSO (2%) were performed. Tested concentrations were 1500 to 5.85 μg·mL^−1^. After 24 h of incubation, the plates were then examined under a binocular microscope Lupa (X3.0), and the numbers of live nauplii in each well were counted to determine the medium lethal concentration (LC_50_) [34]. Assays were performed in three independent studies.

In all toxicity assays, DMSO (Sigma-Aldrich^®^) at 2% and NLS were used as a control.

## 4. Conclusions

This study demonstrated that the incorporation of copper(II) complexes containing isoniazid as a ligand in their coordination sphere into nano-sized drug delivery systems increased their antimycobacterial activity, decreased cytotoxicity against the Vero cell line, and consequently improved the selectivity index up to 170 times higher in some instances, based on *in vitro* analyses. The toxicity in an alternative model also showed a reduction after the incorporation of the compounds into the nanosystem. Such a nanosystem can be a vehicle for the delivery of drugs against *Mycobacterium tuberculosis*.

## Figures and Tables

**Table 1:** Droplet size and polydispersion index of the nanostructured lipid system (NLS) and copper(II) complexes loaded into the NLS. Each value represents the average (±standard deviation) of three repetitions.

Formulation	Mean Diameter ± S.D. (nm) *	Mean PDI ± S.D. *
NLS	169.5 ± 0.7095	0.135 ± 0.0130
1-Loaded	211.1 ± 0.8963	0.200 ± 0.00900
2-Loaded	205.5 ± 1.652	0.226 ± 0.0150
3-Loaded	209.8 ± 0.8083	0.236 ± 0.00100

* Standard deviation (S.D.); polydispersity index (PDI).

**Table 2:** Zeta potential of the NLS and copper(II) complexes loaded into the NLS. Each value is the average (±standard deviation) of three repetitions.

Formulation	ζ Potential ± S.D. (mV) *
NLS	−0.100 ± 0.103
1-Loaded	−0.0433 ± 0.214
2-Loaded	−0.00690 ± 0.0896
3-Loaded	−8.43 ± 1.63

* Standard deviation (S.D.).

**Table 3:** Flow index (η) and consistency (K) of NLS, 1-, 2-, and 3-loaded into the NLS.

Formulations	η	K
NLS	0.45	1.09
1-Loaded	0.20	9.41
2-Loaded	0.37	4.73
3-Loaded	0.20	9.57

**Table 4:** Results of biological assays (MIC and IC_50_) and determination of SI of the free copper(II) compounds and those loaded into the NLS against *Mtb* H_37_Rv.

Complexes	MIC (μg·mL^−1^)	IC_50_ Vero (μg·mL^−1^)	SI	IC_50_ J774.A1 (μg·mL^−1^)	SI	IC_50_ MRC-5 (μg·mL^−1^)	SI
1-Not loaded	20.46	109.5	5.351	80.11	3.915	76.80	3.754
1-Loaded	0.3970	367.6	926.0	10.47	26.37	9.120	22.97
2-Not loaded	5.913	314.3	53.15	75.36	12.74	51.90	8.777
2-Loaded	0.2190	>500.0	2283	68.48	312.7	11.77	53.74
3-Not loaded	1.459	325.3	223.0	184.0	126.1	132.0	90.47
3-Loaded	0.3130	>500.0	1597	66.13	211.2	22.82	72.90

**Table 5:** Lethal concentration (LC_50_) determined for free copper(II) compounds or those loaded into the nanosystem in *Artemia salina* L.

Complexes	LC_50_ (μg·mL^−1^)
1-Not loaded	17.10
1-Loaded	297.7
2-Not loaded	7.800
2-Loaded	378.8
3-Not loaded	244.1
3-Loaded	479.2
DMSO 2%	>1500
NLS	170.0
